# Patient Experiences and Insights on Chronic Ocular Pain: Social Media Listening Study

**DOI:** 10.2196/47245

**Published:** 2024-02-15

**Authors:** Brigitte Sloesen, Paul O'Brien, Himanshu Verma, Sathyaraj Asaithambi, Nikita Parashar, Raj Kumar Mothe, Javed Shaikh, Annie Syntosi

**Affiliations:** 1 Novartis Pharma NV Vilvoorde Belgium; 2 Novartis Ireland Limited Dublin Ireland; 3 Novartis Healthcare Pvt Ltd Hyderabad India; 4 Novartis Pharma AG Basel Switzerland

**Keywords:** chronic ocular surface pain, patients' experiences, quality of life, social media, Twitter, unmet needs, ocular pain, ophthalmology, ocular, listening, experience, experiences, tweet, eye pain, eye condition, social media platforms, social media use, patient experience, chronic pain, pain, internet, eye, retina, online health, digital health, web, vision, optical

## Abstract

**Background:**

Ocular pain has multifactorial etiologies that affect activities of daily life, psychological well-being, and health-related quality of life (QoL). Chronic ocular surface pain (COSP) is a persistent eye pain symptom lasting for a period longer than 3 months.

**Objective:**

The objective of this social media listening study was to better understand COSP and related symptoms and identify its perceived causes, comorbidities, and impact on QoL from social media posts.

**Methods:**

A search from February 2020 to February 2021 was performed on social media platforms (Twitter, Facebook, blogs, and forums) for English-language content posted on the web. Social media platforms that did not provide public access to information or posts were excluded. Social media posts from Australia, Canada, the United Kingdom, and the United States were retrieved using the Social Studio platform—a web-based aggregator tool.

**Results:**

Of the 25,590 posts identified initially, 464 posts about COSP were considered relevant; the majority of conversations (98.3%, n=456) were posted by adults (aged >18 years). Work status was mentioned in 52 conversations. Patients’ or caregivers’ discussions across social media platforms were centered around the symptoms (61.9%, n=287) and causes (58%, n=269) of ocular pain. Patients mentioned having symptoms associated with COSP, including headache or head pressure, dry or gritty eyes, light sensitivity, etc. Patients posted that their COSP impacts day-to-day activities such as reading, driving, sleeping, and their social, mental, and functional well-being.

**Conclusions:**

Insights from this study reported patients’ experiences, concerns, and the adverse impact on overall QoL. COSP imposes a significant burden on patients, which spans multiple aspects of daily life.

## Introduction

Chronic ocular surface pain (COSP) is defined as moderate to severe corneal-induced chronic pain lasting more than 3 months. COSP distracts from, or interferes with, regular daily activities, which may result in poor quality of life (QoL) and health-related QoL, thus impacting the individuals’ ability to carry out daily activities and their psychological well-being [1]. Common pain-related symptoms include ocular irritation, burning, dryness, and other pain descriptors such as corneal sensitivity, shooting, stabbing, grittiness, sharp pain, a hot burning sensation, light sensitivity, and the sensation of a foreign body on the ocular surface. The intensity of ocular pain ranges from simple discomfort to intense unbearable pain; however, evidence elaborating a classification system for COSP is limited [2,3]. COSP can be considered an umbrella term due to the wide spectrum of clinical manifestations, which can result from multiple factors [4].

Patient-reported outcomes (PROs) and patients’ experiences are most often analyzed via conventional methods by using cross-sectional surveys (digital, face-to-face interviews, questionnaires, etc) in different research settings (at hospital sites, home, on the web, through patient-supported organizations, etc). In countries with high internet penetration, access to electronic devices and social media platforms has significantly influenced the health care landscape [5,6]. Social media platforms enable patients to share their perceptions about diseases, treatment patterns, satisfaction with outcomes, and other factors affecting their lives. Therefore, these platforms act as a source to obtain disease-related information, identify and access health care resources, network with fellow patients, and communicate problems and experiences on the web [5-7].

Social media listening (SML) is a new approach to gather information from social media platforms and can be useful in generating insights from users’ experiences. SML has been used to monitor and analyze discussions on health-related topics in diverse diseases [8-13]. Social media facilitates efficient capture of patients’ understanding of a disease and their coping strategies. In addition, SML provides a clear understanding of the patients’ perspectives of a disease, their barriers to health behavior change, and disease-related symptoms [14]. To date, there is no published literature on the use of SML to investigate the needs and experiences of patients with COSP. This study explores SML as a research tool to provide insights on disease burden, diagnosis, treatment patterns, and QoL in patients with COSP.

The objectives of this study are to (1) understand the burden of COSP; its symptoms; its impact on daily life, activities, and QoL, including social well-being and mental health; its management; and the hurdles, gaps, and needs from a patient’s perspective; and (2) capture information directly from patients or caregivers by using their own words and in their familiar environment regarding when and how they feel at that moment, attempting to understand related emotions as well.

The results of this study could help define COSP-related gaps and needs from patients’ perspectives and set priorities for drug development to address them. Our results would also help to develop adequate tools (PRO questionnaires) to quantify the impact of COSP on patients and to develop new PRO end points in clinical studies assessing the potential improvement of new treatments.

## Methods

### Study Design and Data Source

A comprehensive search from February 2020 to February 2021 was performed on social media platforms (Twitter, Facebook, blogs, and forums) for English-language content posted on the web. Social media platforms that did not provide public access to information or posts were excluded. The following predefined search terms were used to retrieve the relevant web content: “chronic ocular pain” OR “chronic eye pain” OR “ocular surface pain” OR “ocular pain” OR “eye pain” OR “eye aching” OR “eye ache” OR “eye surface pain” OR “keratoconus pain” OR “conjunctivitis pain” OR “keratitis pain” OR “blepharitis pain” OR “Iritis pain” OR “corneal neuropathy pain.” Social media posts from Australia, Canada, the United Kingdom, and the United States were retrieved using the Social Studio platform [15]—a web-based aggregator tool. In the first step (wave 1), the study was limited to English-speaking countries; non–English-speaking countries were assessed later (in wave 2) and will be the subject of a separate publication.

### Selection of Posts and Text Data Cleaning

We used the Social Studio tool to download the links of posts with the date stamp and geographic location of users from web-based social media platforms on the basis of the abovementioned keywords. The tool assigned a unique article ID for each downloadable link. These links were used to retrieve the content and remove duplicates (based on unique article IDs and content snippets), irrelevant comments, and out-of-scope content. Irrelevant posts including job postings, aggregator, or junk websites (ie, websites leading to unsolicited advertisements or unrelated misleading links), nonfunctional links, promotional content, and pharmaceutical or e-retailer market reports were removed. Spelling correction was applied while evaluating the content of the post. Additional secondary research including hand searching for posts was performed for patients’ or caregivers’ conversations on forums to increase the robustness of insights and volume of data. The term “mention” indicates the number of times a symptom, treatment, diagnostic test, or another parameter is mentioned in each post. The number of mentions were independent of the number of posts, and due to data anonymization, these users could not be tracked across different platforms. The terms “conversation,” “post,” and “discussion” were synonymously used.

### Categorization and Indexing of Social Media Posts

**Figure 1:**
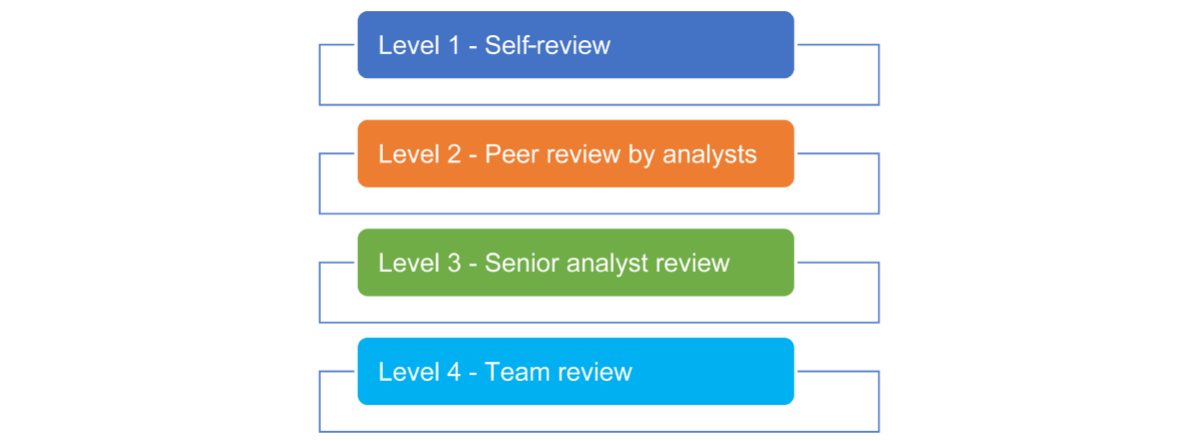
Decision-making process for including and tagging of posts.

The downloaded links were further indexed using the WordNet Lemmatizer natural language processing (NLP) model to arrive at a sample of possible patient posts using patient lexicons and disease-related keywords. A relevancy check was carried out through a two-step process: (1) NLP was used to identify the relevancy and (2) a manual evaluation of relevant posts identified using NLP was carried out. Gender, age, work status, and other demographic information about the users was recorded when available or categorized as unknown if the information was not available at the time. Final analysis was conducted manually to generate patient or caregiver insights. A 4-level hierarchy of the decision-making process was followed to ensure the creation of a robust model of consensus-based analysis and tagging of posts. A self-review, followed by a peer review by analysts, a review by senior analysts, and finally a team review were performed during the study (Figure 1).

The key themes identified were patient demographics, perceived causes of COSP, symptoms, disease management (medical consultations, diagnosis, and treatments), and QoL aspects (impact on physical, functional, emotional and social well-being, and work productivity). Posts were further analyzed for the “number of mentions” of a particular theme in the posts. Posts with a mention of COSP-related symptoms (itching, pain, irritation, etc) were used to provide a qualitative description of the patients’ self-reported experience with the disease. The impact of COSP on patients’ QoL was analyzed using posts describing work productivity loss or inefficiency and problems in performing activities of daily living. One post can have multiple mentions (themes) across the patient journey.

### Ethical Considerations

This paper does not contain any studies with identifiable participants. All web-based content was anonymized and was in accordance with the HIPAA (Health Insurance Portability and Accountability Act) search strategy and data collection. Approval was obtained from the Novartis safety registry 1P1R—the governing body that holds oversight on the use of social media by Novartis (1P1R ID DE005899). All relevant local and global laws affecting and relating to the use of social media were aligned with and, as reflected in Novartis processes, followed in the conduct of this study. The authors of this manuscript consent to the publication of the submitted manuscript and declare that no individual patient data requiring consent has been presented.

### Data Analysis

All data were analyzed using descriptive statistics and are presented as the number of posts, number of mentions, or percentages. All posts were analyzed by (1) social media channel (Twitter, Facebook, forums, and blogs), (2) country (Australia, Canada, the United Kingdom, and the United States), (3) tone (positive, neutral, or negative), and (4) key themes of discussion.

## Results

### Characteristics of Analyzed Posts

**Figure 2:**
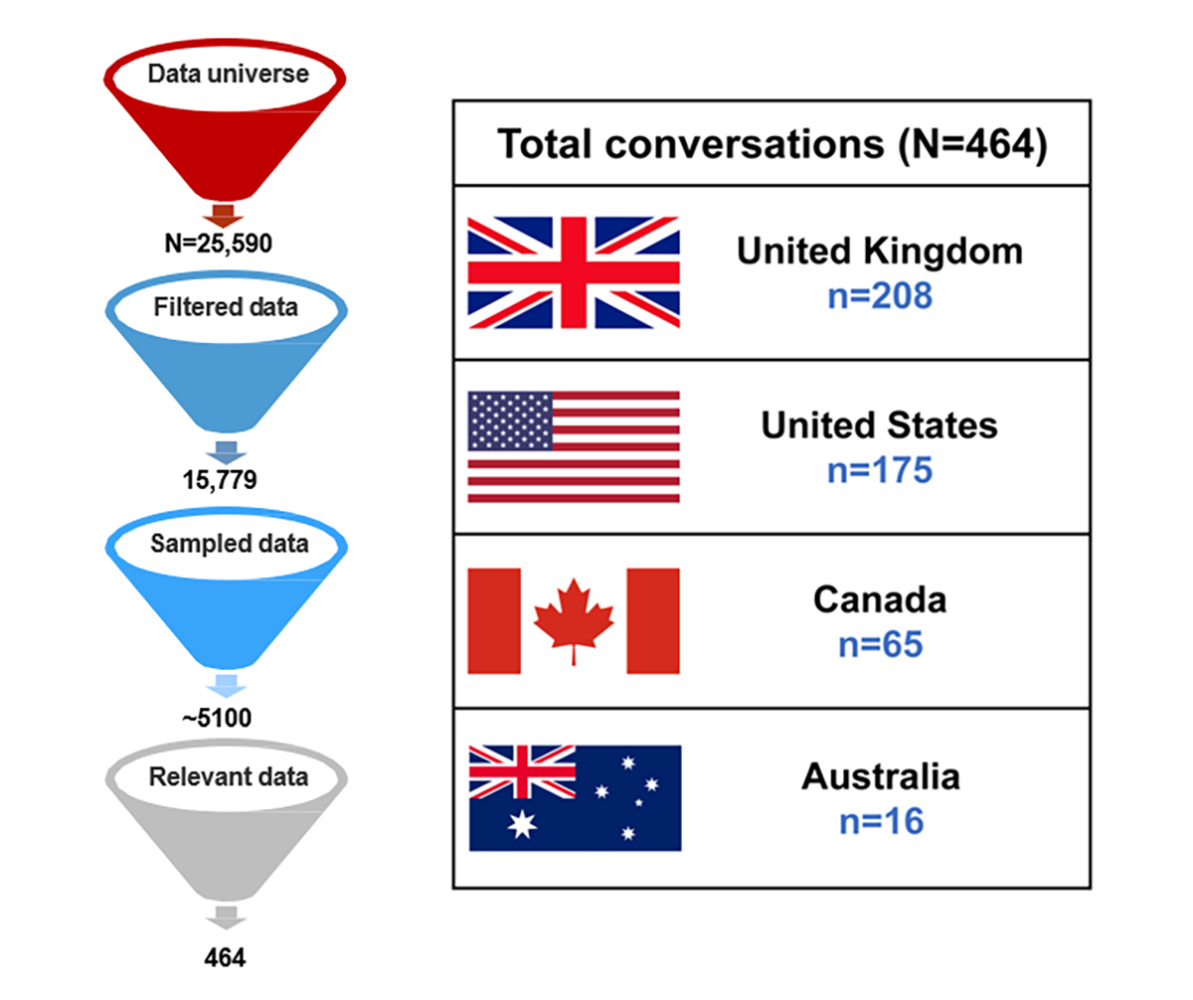
Flowchart for the selection of and tagging of conversations. "N" denotes the number of posts remaining after each step and country-wise conversations data.

A total of 25,590 outputs were initially extracted from social media platforms using COSP-related keywords during the study period. In all, 5100 patient posts were identified by NLP, which facilitated an automated process of generating unique patient posts. The 5100 posts were then filtered for relevance to the disease area by analysts by manually identifying the content that explicitly stated information about COSP. A total of 464 posts determined to be written by patients or caregivers were thus identified and included for further analysis. Figure 2 summarizes the filtering of posts.

Forums and Twitter were the key channels for discussions across all geographies, contributing to ~59% and ~39% of the total conversations, respectively. A detailed list of domains from where the posts were obtained and demographic information are provided in Multimedia Appendix 1. Most of the social media posts were from the United Kingdom (n=208), followed by the United States (n=175), Canada (n=65), and Australia (n=16; Figure 2). Of the 130 conversations mentioning the duration of their ocular pain, the majority of patients had experienced ocular pain for several years (53.1%, n=69), followed by 7 to 12 months (18.5%, n=24), 4 to 6 months (14.6%, n=19), and 3 months (13.8%, n=18).

The majority of conversations were from patients (95%, n=441) as compared to 3.9% (n=18) from caregivers. The gender of the patient was mentioned in 118 posts, of which 57.6% (n=68) of posts were from women and 42.4% (n=50) of them were from men. Only 42 posts specified the patients’ ages; 40.5% (n=17) of patients were 21-30 years of age and 26.2% (n=11) of them were >60 years of age. Approximately 214 posts mentioned whether the patients were adults (99.1%, n=212) or children or teenagers (<18 years of age; 0.9%, n=2). Regarding the work status of the patients determined from among 52 posts, the majority of patients were working (51.9%, n=27), 19.2% (n=10) of them were students, and 19.2% (n=10) of them were retired.

### Key Themes of Discussion

Of the 464 conversations, symptoms (61.9%, n=287) and the causes of COSP (58%, n=269) were the most common themes that emerged, along with the impact on QoL (20%, n=93). Patients or caregivers also discussed their experiences with health care professionals (HCPs; 25%, n=116), diagnosis (8%, n=37), triggers of their COSP (19%, n=88), treatments (18.1%, n=84), and coping strategies and lifestyle modifications (15.9%, n=74).

**Figure 3:**
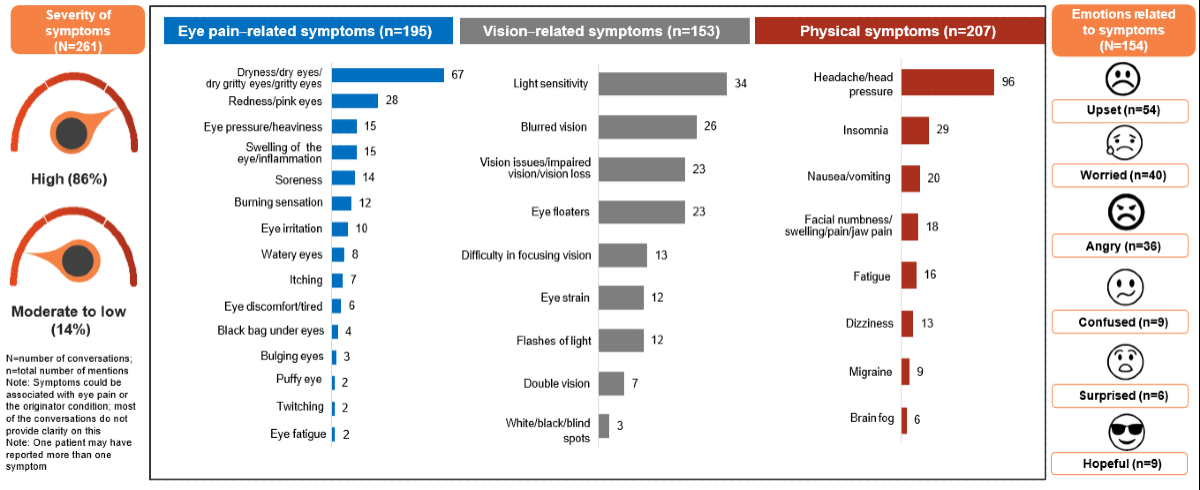
Patient-reported symptoms on social media by the number of mentions in the posts.

Symptoms described by patients varied widely and could be categorized as eye pain–related, such as burning and irritation; vision-related, such as blurred vision and light sensitivity; and physical, such as insomnia and headache (Figure 3). The severity of the symptoms was ranked (high and moderate to low) on the basis of key words and phrases used in the posts. Of the 261 conversations, 85.8% (n=224) of posts were rated as high severity and 14.2% (n=37) as moderate to low severity. Some phrases for high severity mentioned by patients included “eyes burn like hell,” “excruciating (eye) pain,” “eye pain is the absolute worse,” “unbearable pain,” etc. Moderate to low–severity phrases included “mild redness in my eye occasionally,” “discomfort to mild pain,” and “the pain is pretty manageable.” In 154 conversations, patients also cited emotions that were related to their symptoms, including being upset (35.1%, n=54), worried (26%, n=40), angry (23.4%, n=36), and confused (5.8%, n=9; Figure 3).

There were 282 mentions of the causes of COSP, of which 46.1% (n=130) mentioned underlying ocular medical conditions such as dry eye disease and ocular surgery, and 53.9% (n=152) of them mentioned nonocular conditions contributing to COSP, such as migraine and COVID-19 (Multimedia Appendix 2). Commonly reported environmental triggers of eye pain, such as exposure to bright light or sun and long screen time use (Multimedia Appendix 3), reflect the burden on daily activities.

Overall, 158 patients mentioned the type of HCP they consulted. Of them, 27 consulted an ophthalmologist; 25 consulted an eye, retina, or cornea specialist; 21 consulted a doctor (unspecified specialty); 15 consulted an optometrist; and 14 consulted an optician. Others consulted a neurologist, neuro-ophthalmologist, eye clinic or eye surgeon, otorhinolaryngologists, etc. When the HCP consulted was mentioned (158 patients), the majority of the related comments (n=123) were negative. Many patients expressed that they were not able to get a proper diagnosis and hence expressed negative sentiments in various conversations, citing emotions such as confusion and worry. Pharmaceutical drugs (140/247, 56.7% treatment-related mentions) were cited as the major treatment option for managing COSP symptoms such as topical or oral drugs, followed by alternative remedies (83/247, 33.6%) such as hot compresses, lenses, etc, and lifestyle modifications (24/247, 9.7%) such as sleep, diet changes etc (Multimedia Appendix 4).

### Impact of COSP on Patients’ QoL

**Figure 4:**
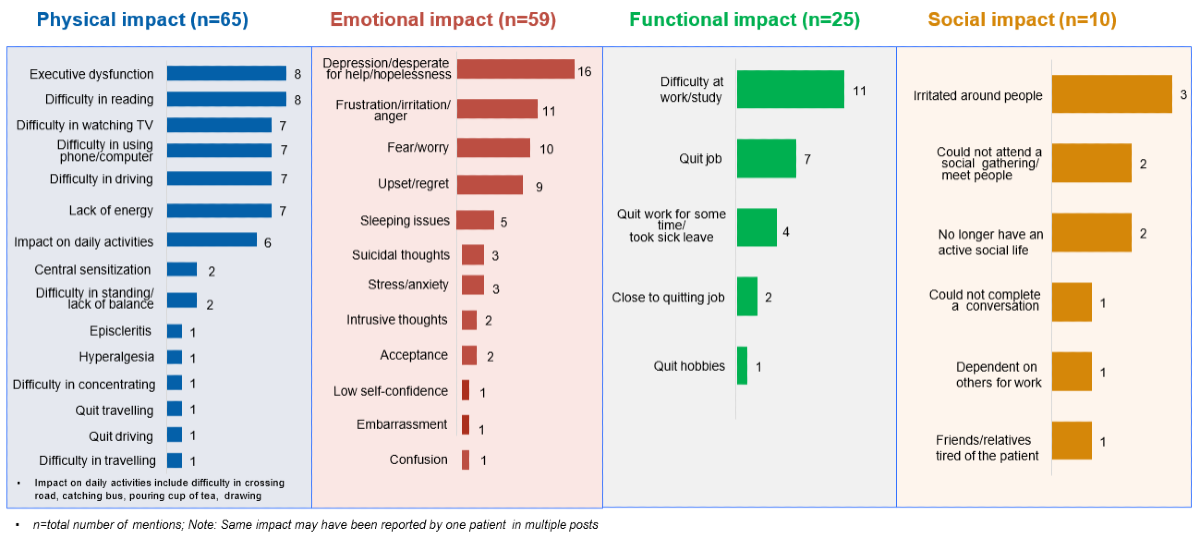
Impact of chronic ocular pain on patients’ quality of life.

**Figure 5:**
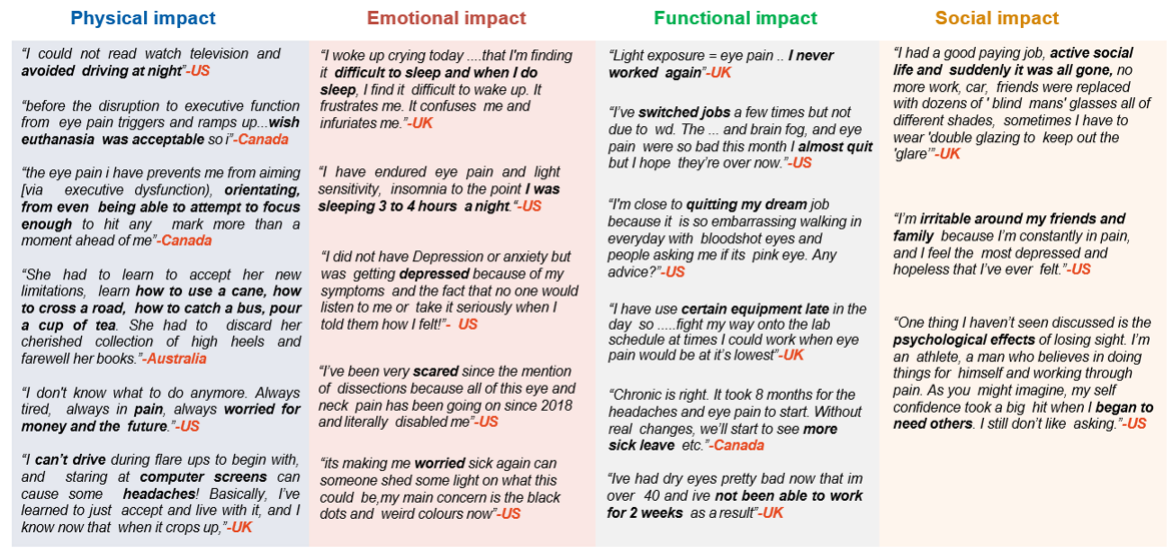
Patients' and caregivers' example quotes regarding quality of life.

A total of 159 posts by patients, ascertained to describe their QoL, were grouped in 4 domains: physical, emotional, functional, and social impact (Figure 4). COSP symptoms significantly impacted all aspects of patients’ QoL. Of the 159 posts, various aspects reported were difficulty watching TV or using a phone or computer (8.8%, n=14), executive dysfunction (cognitive, behavioral, and emotional difficulties; 5%, n=8), difficulty performing day-to-day activities such as reading (5%, n=8), and difficulty driving (4.4%, n=7). Emotional well-being, including feelings of depression or hopelessness (10.1%, n=16), frustration or anger (6.9%, n=11), fear (6.3%, n=10), and even suicidal thoughts (1.9%, n=3), were also mentioned. Conversations also mentioned the impact of COSP on functional well-being such as difficulty at the work place or at the place of study (6.9%, n=11), reduced productivity or having to quit their job (5.7%, n=9), as well as social impacts such as being irritated around people (1.9%, n=3) and having a less active social life (2.5%, n=4). Overall, COSP greatly affected the patients’ functional and psychological well-being, as illustrated through patients’ example quotes in Figure 5.

### Patients’ Perspectives on Unmet Needs

**Figure 6:**
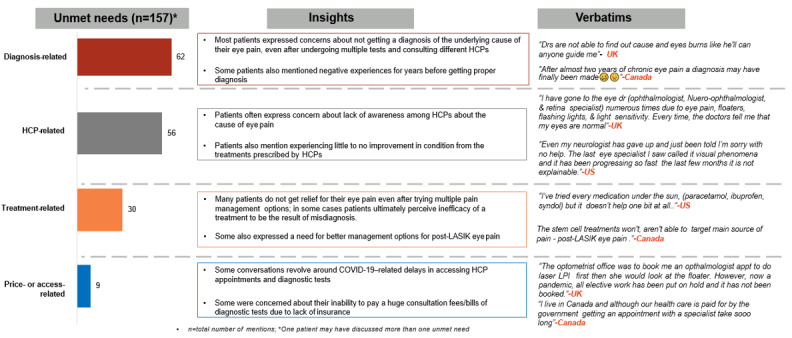
Key unmet needs across different themes. HCP: health care professional.

Patients’ perceptions of unmet needs were grouped under 4 broad categories: diagnosis, HCP-related, treatment, and price- or access-related. A summary of patients’ perceptions of unmet need is presented in Figure 6. Failure to obtain a diagnosis of the underlying cause of COSP, even after undergoing multiple tests, emerged as the primary concern; this also led to a loss of confidence in HCP consultations in most cases.

Although not assessed in detail, no major differences were observed among the 4 English-speaking countries. Of note, however, the eye pain and related symptoms described by patients most often referred to the eye in general without specification of the ocular surface, so the study assessed chronic ocular pain rather than COSP.

## Discussion

### Principal Findings

This SML study attempts to use relevant data from social media platforms to understand patients’ problems and concerns related to COSP. This is the first study to use an SML approach to analyze the COSP disease burden and patients’ perspectives on COSP-related symptoms, causes and triggers, treatment options, and QoL aspects. The study provides a useful insight of the significant burden of COSP on patients’ daily activities, such as reading and driving and the negative impact on their emotional and social well-being and their work productivity. The strong wording that patients are using in describing their pain and its impact on their daily life illustrates patients’ suffering as well as their frustration in desperately seeking effective relief for their chronic ocular pain. This highlights the importance and difficulty of interpreting patients’ own wording expressed in spontaneous SML quotes to understand their real suffering. The results also illustrate how the lack of disease awareness to confirm a diagnosis, a dearth of effective treatment options, and the need to increase awareness of COSP among patients and HCPs are the key unmet needs.

The available treatment options for chronic eye pain are very limited, ranging from the use of artificial tear drops, anti-inflammatory agents or local immunomodulators, and analgesic drugs [16]. In this study, symptoms, multifactor etiological causes, diagnosis, and especially treatments of COSP are most frequently discussed in the context of their impact on patients’ lives. This is suggestive of the need for current COSP treatment paradigms to include qualitative aspects of patient experience to develop a patient-centric model of COSP management.

Two broad aspects of patient experience that this study identify are negative impact on QoL and patient perception of unmet needs. Almost 1 in 5 COSP patients described the negative impacts of COSP on work performance. Treatment strategies could be improved by understanding the issues related to patients’ QoL from social media posts. A common theme among patients’ conversations was the delay in the diagnosis of COSP, which resulted in multiple clinic visits and suboptimal management of COSP symptoms. In this study, patients reported that delayed diagnosis and a lack of proper treatments are associated with a negative perception of COSP. Additionally, even after being diagnosed with COSP, patients cited a lack of pain relief despite using multiple pain management options.

Social media interactions on the web present an inherent opportunity to identify and analyze unfiltered experiences of COSP directly from the own words of patients and caregivers. SML has been shown to be a valuable approach to gain an understanding of the patients’ experience of diseases and treatments with a particular advantage that it can allow data to be gathered from a very large, representative sample that is geographically dispersed (ie, from a wide range of countries or locations) [17]. SML has been used as a tool to investigate patients’ perceptions for different indications, including cancer screening [9], dry eye disease [18], HIV [11], inflammatory bowel disease [10], multiple sclerosis [8], presbyopia [19], total joint arthroplasty [13], and Zika virus [12]. Insights and summaries derived from such conversations can be a vital resource in enriching treatment outcomes associated with chronic diseases. It is, however, critical to carefully detect and assess different aspects included in the sometimes lengthy web-based conversations, ensuring the correct interpretation of patients’ posts, including the underlying emotional aspects. In our study, the high severity of COSP symptoms and its lasting effect resulted in negative sentiments in most patients. The variety in terms and the often strong wording used to describe their pain symptoms, their feelings, and the impact on their daily lives reflects the importance of SML in collecting first-line patient information that has been spontaneously expressed. Careful attention must be paid to capture the different aspects addressed in multiple conversations and interpret them correctly to adequately understand individuals’ actual suffering. Correct categorization and indexing of the SML posts was critical. Multiple analysts, senior analysts, and experts reviewed the posts during the whole process in order to obtain robust data. This highlights the importance and difficulty of interpreting patients’ own wording expressed in spontaneous SML conversations.

NLP has been widely used in SML studies, and the resulting data have been reported for a variety of diseases [10,12]. In this SML review, NLP has been used for its high-throughput capability, which was then paired with manual assessment to provide the necessary focus and specificity of the outputs. Inherent biases that may affect the accuracy (representativeness of the collected sample and linguistic selection in posts), reliability (consistency of reports from individual patients across time points or descriptors), and quality of information (self-selection bias) obtained from any social media platform may be present in this study as well [20]. One of the limitations of this study was that only publicly available information on digital platforms has been accessed and used; all the personal identifiers were anonymized in the report. Further, owing to the unstructured nature of social media data, it was not possible to obtain information on every research question; therefore, we objectively determined whether the available information supported the research question and, accordingly, our findings, which may be considered a limitation of this study.

### Conclusions

Insights from this study reported the experiences and concerns and the adverse impact on overall QoL among patients with COSP. Assessment of patients’ own wording demonstrated that COSP imposes a significant burden of impaired QoL on patients, which spans multiple aspects of daily life, including physical and functional impacts, as well as impacts on emotional and social well-being. The lack of disease awareness among HCPs with long diagnostic delays and the inefficacy of prescribed treatments were cited as concerns. The variety in terms patients used on the web to describe their suffering, issues, and needs as well as the different aspects addressed herein contribute to a better understanding of the patients’ perspectives and highlight the value of SML studies. It is indeed most critical to obtain patients’ insights and address their unmet needs when considering disease management including drug development.

## Appendix

Multimedia Appendix 1 Demographic and clinical characteristics as represented by patient posts.

Multimedia Appendix 2 Patients' and caregivers' example quotes regarding ocular causes of chronic ocular surface pain.

Multimedia Appendix 3 Patients' and caregivers' example quotes regarding nonocular causes of chronic ocular surface pain.

Multimedia Appendix 4 Patients' and caregivers' discussion on social media regarding treatment options for chronic ocular surface pain.

## Abbreviations

COSP: chronic ocular surface pain
HCP: health care professional
HIPAA: Health Insurance Portability and Accountability Act
NLP: natural language processing
PRO: patient-reported outcome
QoL: quality of life
SML: social media listening

## Data Availability

The data sets generated or analyzed during this study are available from the corresponding author on reasonable request.
