# Supplementary material for: Patient Experiences and Insights on Chronic Ocular Pain: Social Media Listening Study
Source: JMIR Form Res. 2024 Feb 15;8:e47245. doi: 10.2196/47245 (PMC10905354; doi:10.2196/47245)
Supplement: Multimedia Appendix 1 [file formative_v8i1e47245_app1.docx]

Multimedia Appendix 1

Demographic and clinical characteristics as represented by patient posts

| **Characteristic** | **Percentage of patients (n/N)** |
| --- | --- |
|  |  |
| **Gender** |  |
|  |  |
| Female | 58 (68/118) |
| Male | 42 (50/118) |
| **Age group (years)** |  |
|  |  |
| 11-20 | 11.9 (5/42) |
| 21-30 | 40.5 (17/42) |
| 31-40 | 11.9 (5/42) |
| 41-50 | 2.4 (1/42) |
| 51-60 | 7.1 (3/42) |
| >60 | 26.2 (11/42) |
| **Audience segmentation** |  |
|  |  |
| Patients | 95 (441/464) |
| Caregivers | 4 (18/464) |
| Unidentified | 1 (5/464) |
| **Work status** |  |
|  |  |
| Working | 52 (27/52) |
| Student | 19 (10/52) |
| Retired | 19 (10/52) |
| Quit job | 7.7 (4/52) |
| Looking for work | 1.9 (1/52) |
| **Online platform segmentation** |  |
|  |  |
| Forum | ~59% |
| Twitter | ~39% |
