# Supplementary material for: Patient Experiences and Insights on Chronic Ocular Pain: Social Media Listening Study
Source: JMIR Form Res. 2024 Feb 15;8:e47245. doi: 10.2196/47245 (PMC10905354; doi:10.2196/47245)
Supplement: Multimedia Appendix 2 [file formative_v8i1e47245_app2.pdf]

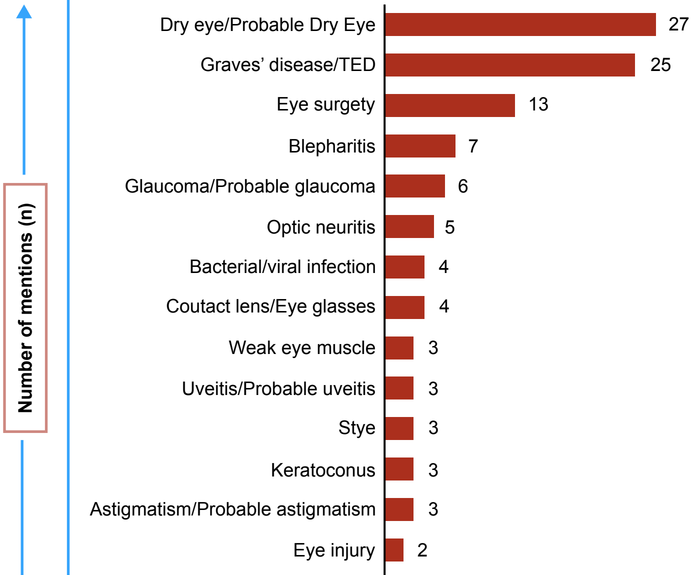

- *"Probable" primarily incluses cases where the patient assumes/suspects to have a disease*
- *TED stands for Thyroid Eye Disease*

Just found out that my shooting eye pain is a mix of ***glaucoma and dry eye****"* ***-Canada***

"I have chronic eye pain due to weak eye muscles, hypersensitivity to light, and ***Severe dry eye****;"* ***-US***

"When your eyes are sensitive to light and cause migraines, but you also can't see well in the dark either. Just being able to see is a balance between pain and necessity ***#keratoconus"*** ***-US***

"Eye specialist diagnosed a lazy eye in childhood, ***astigmatism*** *later. Kept going back to Dr with eye pain"* ***-UK***

"I had ***an eye injury*** *in 1990, 4 days after my eldest son started school. It left me with hemeralopia/photophobia, eye pain is not nice."* ***-UK***

"I have chronic eye pain from nerve damage in my eyes as a result of ***laser eye surrgery****"* ***-Australia***

"I'm bored of having a ***stye prone eye*** *now like. My doctor warned me about this years ago."* ***-UK***
