# Supplementary material for: Patient Experiences and Insights on Chronic Ocular Pain: Social Media Listening Study
Source: JMIR Form Res. 2024 Feb 15;8:e47245. doi: 10.2196/47245 (PMC10905354; doi:10.2196/47245)
Supplement: Multimedia Appendix 3 [file formative_v8i1e47245_app3.pdf]

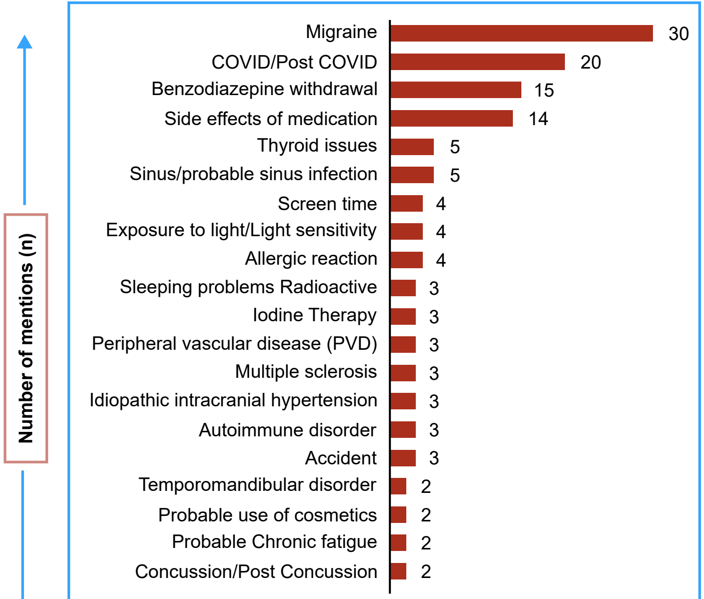

“Probable: includes cases where the patient assumes/suspects they have a disease
Note: Same causes may have been mentioned by one patient in multiple posts
Non-ocular section includes the causes of eye pain which are not eye diseases.

“Anyone else get like 1-2 ***migraines*** *a year that start off with blurry vision then transform into total head pain, eye pain and neck pain then throwing up. ”* ***-Australia***

“I can’t do the things I love most because of ***#COVID****......My eye pain only goes away if I look down at the floor”* ***-Canada***

“Even on ***sinus and allergy meds*** *and saline rinses my right maxillary sinus is swollen and not happy. Everything is right sided — I get pain in my top teeth, pain behind my eye, pain in my temple but also in other parts of my head’* ***-UK***

“At my first appointment with the rheumatologist, I told him that I probanly had ***Sjogren’s Syndrome****. He raised his eyebrows at my self-diagnosis, did the blood work, and agreed with it......Ongoing issues include: GERD and other digestive troubles, overall dryness, eye pain”* ***-Canada***

““I’m really ok until I try reading on ***my computer*** *and then I’m screwed”* ***-US***

“I pushed myself and worked all day and didn’t stop working when the ***eye pain set in at my computer****”* ***-US***

“I’m ***limiting screen time****, so I’ll not be around as much”* ***-Canada***
