# Supplementary material for: Patient Experiences and Insights on Chronic Ocular Pain: Social Media Listening Study
Source: JMIR Form Res. 2024 Feb 15;8:e47245. doi: 10.2196/47245 (PMC10905354; doi:10.2196/47245)
Supplement: Multimedia Appendix 4 [file formative_v8i1e47245_app4.pdf]

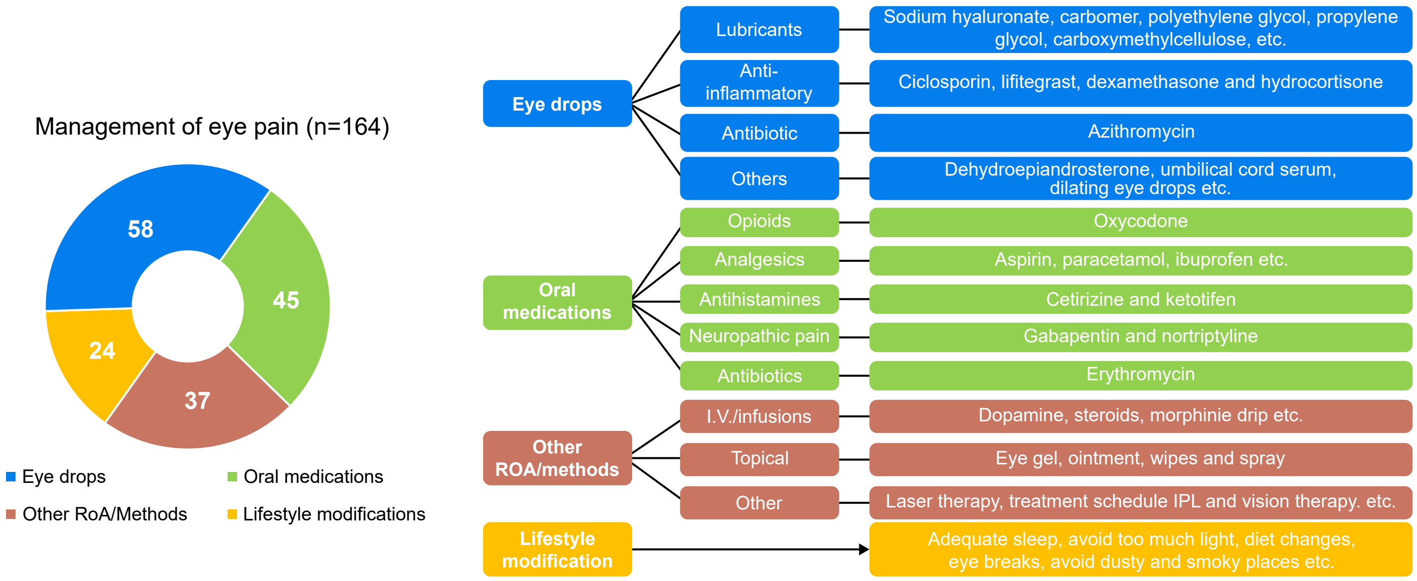

Alternative medicines: Hot compress (n=11), blue light filter glasses/lenses (n=9), eyeglasses (n=8), eye mask (n=6), cold compress (n=2), sunglasses (n=2), etc.

- *n=Indicates total number of mentions; ROA: Route of Administration; IPL: Intense Pulsed Light Therapy; I.V.: intravenous*
